# Covalent Organic Frameworks for Immunoassays: A Review

**DOI:** 10.3390/bios15070469

**Published:** 2025-07-21

**Authors:** Suling Yang, Hongmin Liu

**Affiliations:** Henan Province Key Laboratory of New Opto-Electronic Functional Materials, College of Chemistry and Chemical Engineering, Anyang Normal University, Anyang 455000, China; 15832311979@163.com

**Keywords:** electrochemical immunoassays, optical immunoassays, covalent organic frameworks, electrode materials, signal labels

## Abstract

Immunoassays relying on highly specific antigen–antibody recognition are important tools for effectively measuring the levels of various targets. Efforts have been made in the development of various methods to improve the detection sensitivity and stability of immunoassays. Covalent organic frameworks (COFs), as an emerging class of novel crystalline porous materials, have unique advantages such as flexible designability, high surface area, excellent stability, tunable pore sizes, and multiple functionalities. They have great potential as novel sensory materials. Herein, we summarize the advances of COFs in electrochemical and optical immunoassays serving as electrode modifiers, signal indicators, enzyme or probe carriers, etc. Meanwhile, the design and application of typical COFs-based immunoassays in the determination of different targets are discussed in detail. Finally, challenges and future perspectives are presented.

## 1. Introduction

Immunoassays based on highly specific immunological reactions between antigens and antibodies possess high sensitivity and specificity. They have been developed as important tools for the detection of various targets in complex samples, including small molecules, proteins, enzymes, exosomes, and cells [1]. Typically, enzyme-linked immunosorbent assay (ELISA) is popularly used because of its high specificity and successful commercialization. However, this method suffers from the inherent drawbacks of enzyme labels, such as high production cost, short shelf life, sensitivity to environmental change, and low activity after binding, leading to an increasing demand for more effective alternatives [2]. In order to improve the sensitivity and stability of biosensors, various functional materials have been introduced into the development of immunoassays for signal amplification, such as noble metal nanomaterials, carbon nanomaterials, metal oxide/sulfide nanostructures, metal–organic frameworks and so on [3,4,5].

As an emerging class of crystalline porous materials, covalent organic frameworks (COFs) are constructed by strong covalent bonds between organic monomers with lightweight elements, including C=N, C=C-N, C-N, and B-O bonds. COFs possess unique advantages such as flexible designability, high surface area, excellent stability, tunable pore sizes, and multiple functionalities [6]. Compared with traditional porous materials, such as metal–organic frameworks (MOFs), COFs avoid the use of metal ions, and their functions and topological structures can be precisely designed and predicted. In addition, COFs with robust covalent bonds possess higher chemical and thermal stability than MOFs formed by relatively weak metal coordination bonds [7]. Therefore, COFs have shown remarkable potential in diverse fields, including catalysis, energy storage, gas adsorption, drug delivery, optoelectronics, and sensing [8,9,10,11,12,13,14,15,16]. Recently, COFs have been successfully used to fabricate immunosensors for biosensing applications. Due to their large surface area and abundant functional sites, COFs can be used to immobilize biomolecules through different strategies, including electrostatic interactions, covalent bonds, van der Waals interactions and so on. In addition, COFs with intrinsic optical or electrochemical properties can be directly synthesized by elaborately choosing appropriate organic building blocks. For example, organic molecules containing π-conjugated structures, such as pyrene, triazine, tetraphenylethylene, and triphenylene, can be used to synthesize fluorescent COFs with high photoluminescent quantum yields [17]. Electroactive or metallized organic monomers can be employed to prepare COFs with good redox or electrocatalytic activities [18]. Furthermore, COFs with high surface area and well-defined pores can be combined with other functional molecules and materials to improve sensing capabilities [19]. Therefore, COFs and their hybrids have been recognized as promising candidates to develop various immunoassay platforms.

**Scheme 1:**
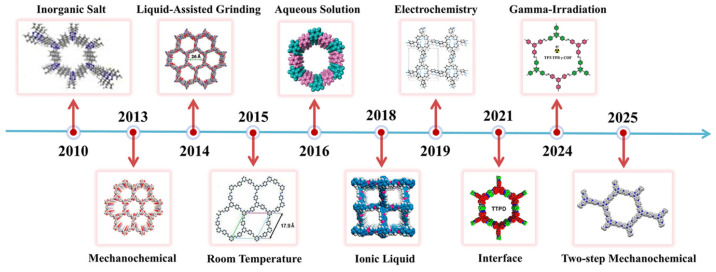
Representative development diagram of the green and large-scale synthesis of COFs. Reprinted with permission from ref. [41]. Copyright 2025 Wiley-VCH.

**Scheme 2:**
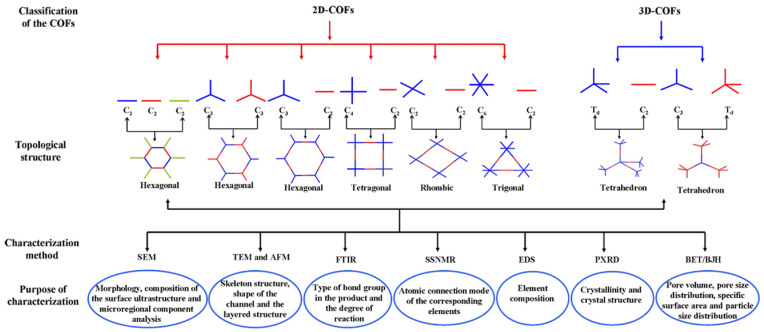
The topological structure, different characterization methods and functions of COFs. Reprinted with permission from ref. [42]. Copyright 2025 Elsevier B.V. SEM, scanning electron microscope; TEM, transmission electron microscope; AFM, atomic force microscope; FTIR, Fourier Transform infrared spectroscopy; SSNMR, solid state nuclear magnetic resonance; EDS, energy dispersive spectrometer; PXRD, powder x-ray diffraction; BET, Brunauer–Emmett–Teller adsorption; BJH, Barrett–Joyner–Halenda mode.

Since the first discovery in 2005, COFs have sparked great research enthusiasm worldwide. Recently, some excellent reviews about COFs-based sensing applications have been reported [19,20,21,22,23,24,25,26]. However, these reviews mainly focus on the synthesis and application of electroactive or fluorescent COFs in chemical sensing. For example, several groups have summarized the latest advances in COFs-based electrochemical and fluorescent sensing methods [27,28,29,30,31,32,33,34,35,36,37]. Although the applications of COFs in different sensing fields have been summarized in the early reviews, only limited chapters involved the progress of COFs-based immunoassays. To the best of our knowledge, there is no specific review to systematically address the development of COFs-based immunoassays. In this review, we mainly focus on the design and application of COFs and their hybrid materials in immunoassays. This review does not provide a detailed introduction to the structure features and synthesis methods of COFs, as interested researchers can read the impressive review papers to find the solutions [17,38,39,40,41,42]. For instance, Zhang et al. reviewed the developments in the green and large-scale synthesis of COFs for practical applications (Scheme 1) [41]. Guo et al. summarized the topological structures and different characterization methods of COFs (Scheme 2) and highlighted their functions and applications in fluorescent sensing [42]. According to the types of signal outputs, COFs-based immunoassays can be classified into electrochemical (electrochemistry, electrochemiluminescence, and photoelectrochemistry) and optical (e.g., fluorescence, colorimetry, surface enhanced Raman scattering or SERS, etc.) methods. To illustrate the role of COFs in immunoassays, several important and typical works are discussed in detail as examples. Finally, the challenges and future perspectives of COFs-based immunoassays are presented.

## 2. COFs-Based Electrochemical Immunoassays

Electrochemical immunoassays have the advantages of low cost, miniaturization, and real-time monitoring [43]. Their selectivity and sensitivity mainly depend on the sensing electrode and signal reporters. According to the format of immunoreaction, electrochemical immunoassays can be designed as direct, competitive, and sandwich methods [44]. In the direct detection mode, the concentration of analyte can be determined based on the target-induced signal change through the antigen–antibody interactions on the sensor surface. Competitive and sandwich methods generally consist of three components: recognition unit, target molecule, and signal output. In order to improve the analytical performance of electrochemical immunoassays, porous materials can be modified on the electrode surface to increase effective surface area, improve electron conductivity, provide catalytic sites, immobilize recognition elements, and/or be used as the carriers to increase the number of signal reporters for each recognition event or directly as the redox probes to generate enhanced electrochemical signals. In this section, the advancements of COFs serving as electrode modifiers and signal labels for electrochemical immunoassays are discussed, respectively.

### 2.1. Electrode Modifiers

**Figure 1:**
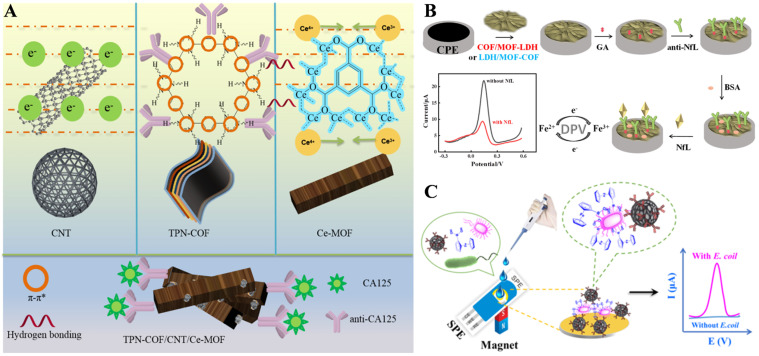
(**A**) Schematic illustration of the CA125 immunosensor based on Ce-MOF/TPN-COF/CNT. Reprinted with permission from ref. [47]. Copyright 2022 Elsevier B.V. (**B**) Schematic illustration of the NfL immunosensor based on COF/MOF-LDH or LDH/MOF-COF. Reprinted with permission from ref. [48]. Copyright 2024 Elsevier B.V. (**C**) Detection mechanism of the *E. coli* immunosensor based on m-COF@IgY and FBA. Reprinted with permission from ref. [50]. Copyright 2022 Elsevier B.V.

Electrode materials can be modified on the sensor surface by electrodeposition, physical adsorption, polymerization, and chemical bonding [45,46]. Recent advancements have provided powerful new strategies for manufacturing COFs-based composite materials as electrode modifiers, further improving detection sensitivity by enlarging electrode surface area and enhancing electron conductivity (Table 1), including MOF/COF [47,48,49], magnetic nanoparticle (MNP)/COF [50], metal nanoparticle/COF [51,52,53,54,55,56,57], and graphene/COF [58]. Crystalline porous materials, including MOFs and COFs, have aroused great interest in the design of electrode materials due to their inherent structural characteristics, such as rapid ion transport, ultra-high and adjustable porosity, large surface area, and abundant redox metal centers [59]. Their composite materials with other nanostructured materials, such as graphene, carbon nanotube (CNT), metal nanoparticle, metallic oxides, and conductive polymers, have a significant impact on the electrochemical performances of sensing devices [60]. The combination of COFs and MOFs enables the hybrids with the advantages of larger specific surface area, richer pore structure, and higher biological affinity for immunocomplexes [61,62,63]. In addition, the MOFs/COFs hybrids exhibit strong affinity with immunocomplexes through electrostatic, π-π stacking, and hydrogen-bonding interactions, which can improve electrochemical sensing capabilities. For this view, An et al. developed a label-free immunosensing platform for carcinomicantigen 125 (CA125) detection by integrating terephthalonitrile-based-COF (TPN-COF) and CNT into Ce-MOF (Figure 1A) [47]. The Ce-MOF/TPN-COF/CNT hybrids had abundant active sites and excellent electronic conductivity. The hydrogen bonds formed between the N atoms in the triazine ring of TPN-COF and the amino and carboxyl groups allowed for the immobilization of more antibodies. The abundant carboxyl groups in trimesic acid and the unsaturated Ce^3+^ ions in porous Ce-MOF improved electron transfer rate. The hybrids effectively amplified the electrochemical signal and facilitated the direct detection of CA125 with a detection limit down to 0. 088 mU/mL. In addition, Li et al. reported the detection of neurofilament light chain (NfL) using COFs-based hybrids as the electrode materials, which were prepared by the integration of polyvalent Tb-MOF and CoAl-layered double hydroxides (CoAl-LDH) with triazine-COF by a hydrothermal method (Figure 1B) [48]. The LDH/MOF-COF-modified electrode was used to determine NfL with a detection limit of 17 fg/mL, providing a promising method for early diagnosis of Alzheimer’s disease. In this work, the used triazine-COF has excellent pore structure, while LDH can produce a large number of small bubbles during the synthesis process due to the degradation of urea, resulting in more small pores in LDH/MOF-COF and achieving excellent detection performances. In addition, COFs can be modified on the surface of paramagnetic materials for the immobilization of antibodies and other functional biomolecules, allowing for magnetic immunosensing platforms. For example, Xiao et al. reported the detection of *Escherichia coli O157:H7* (*E. coli*) by using magnetic COF (m-COF) to immobilize egg yolk antibody (IgY) for the capture of *E. coli* with high specificity and affinity (Figure 1C) [50]. The redox probe of ferrocene boronic acid (FBA) was attached on the m-COF@IgY-*E.coli* surface through the interactions between boronic acids and cis-diol-containing molecules on the *E. coli* surface. The resulting m-COF@IgY-*E.coli*/FBA sandwich complexes conjugates were captured by the magnetic screen-printed electrode to produce a redox signal from the electrochemical oxidation of FBA. The method achieved detection of *E. coli* in the range of 10–10^8^ CFU/mL with a detection limit of 3 CFU/mL and was used for the evaluation of food samples with satisfactory results.

**Figure 2:**
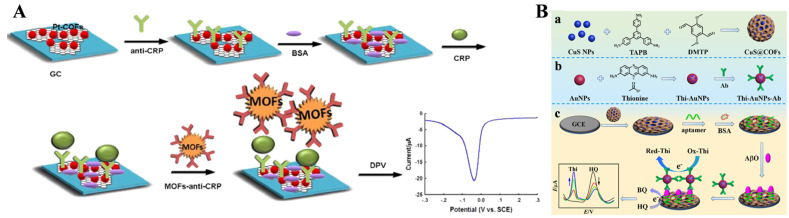
(**A**) Schematic illustration of the CRP immunosensor. Reprinted with permission from ref. [52]. Copyright 2016 American Chemical Society. (**B**) Schematic illustration for the preparation of CuS@COFs (**a**) and Thi-AuNPs-Ab bioconjugates (**b**) and the fabrication of ratiometric electrochemical immunosensor (**c**). Reprinted with permission from ref. [53]. Copyright 2022 American Chemical Society.

Sandwich immunosensors consist of three core events: molecular recognition, target binding, and signal readout. The excellent thermal/chemical stability and good biocompatibility of COFs can facilitate the immobilization of biomolecules through hydrogen-bonding, electrostatic, and covalent interactions. In addition to being used as the electrode materials for direct immunoassays, COFs can also be used as the electrode modifiers for sandwich immunoassays due to their high porosity, large surface area, and low density. Liu et al. reported the electrochemical immunoassay of CRP using platinum nanoparticle-modified COFs (Pt-COFs) as the electrode modifiers to immobilize capture antibody anti-CRP (Figure 2A) [52]. In this work, Cu-based MOFs were used as signal labels based on the electrochemical reduction of Cu^2+^ ions. Pt-COFs improved the sensitivity of electrochemical immunoassays because the periodic arrays of π clouds in the layered structure of two-dimensional COFs can promote charge-carrier transport. This is the first report using Pt-COFs as the substrates for the design of electrochemical immunoassays. In addition, Chen et al. reported a dual-signal ratiometric strategy for the sandwich electrochemical immunoassay of amyloid-β oligomer (AβO) using small copper sulfide nanoparticle-engineered COFs (CuS@COFs) hybrids as both the electrode modifiers and electrocatalysts (Figure 2B) [53]. Herein, electroactive thionine (Thi) and anti-AβO-modified gold nanoparticles (Thi-AuNPs-Ab) were used as additional signal reporters. The CuS@COFs could catalyze the electrochemical oxidation of hydroquinone (HQ). After the capture of AβO and Thi-AuNPs-Ab, the electrochemical signal of HQ decreased while that of Thi increased, achieving the ratiometric electrochemical detection of AβO with a detection limit of 0.4 pM. This method can effectually eliminate background signal drift, thereby avoiding external interference and improving detection precision and clinical reliability for determining the levels of AβO in real cerebrospinal fluid samples.

**Figure 3:**
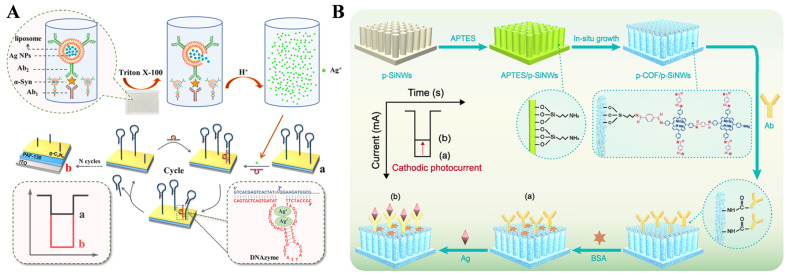
(**A**) Schematic illustration of the liposome-mediated split-type PEC sensing mechanism for α-Syn. Reprinted with permission from ref. [66]. Copyright 2021 American Chemical Society. (**B**) Schematic diagram of the photocathodic immunoassay based on an S-scheme p-COF@p-SiNW heterojunction toward cTnI sensing. Reprinted with permission from ref. [67]. Copyright 2023 American Chemical Society.

Photoelectrochemical (PEC) immunoassays have great potential in biological analysis due to their combination of excellent specificity of immunoreactions and excellent sensitivity of PEC techniques [64]. Signal amplification plays an important role in the development of highly sensitive PEC immunoassays. The signals of PEC biosensors mainly come from photoactive materials. To date, inorganic semi-conductive materials are the most prevalent photoactive materials for PEC biosensors. However, the poor adjustability and biological toxicity of inorganic materials limit their wide applications in biosensing fields. The band gap and optical/electronic properties of COFs can be well adjusted through appropriate structural design. Due to their high thermal and mechanical stability, good biocompatibility and tunability, and highly ordered crystalline structures, COFs are believed to be ideal candidates for high-performance visible light-active PEC materials [65]. Several tentative works on COFs-based PEC immunoassays have emerged recently. For example, Gao et al. reported a PEC immunoassay platform for the detection of α-Synuclein (α-Syn) using a two-dimensional pyrene COFs-based photocathode (PAF-130/g-C_3_N_4_) (Figure 3A) [66]. The signal was amplified by liposome-controlled release Ag-activated DNAzyme cycle on the photocathode. In this work, polyclonal anti-α-Syn (Ab_2_) was covalently linked on the surface of AgNPs-loaded liposomes to form Ab_2_-ALL bioconjugates. The bioconjugates were captured by capture antibody Ab_1_-covered 96-well plates through sandwich immunoreactions in the presence of α-Syn. After lysis treatment and acidolysis, a large number of Ag^+^ ions were released. Then, Ag-activated DNAzyme initiated the cleavage recycle of hairpin DNA (HDNA) probes that were pre-immobilized on the electrode surface, leading to effective signal amplification in the photocurrent. The method could determine α-Syn with a detection limit down to 3.6 fg/mL. Differing from generally used amplification strategies, this work focused on the combination of liposome immunoassay with simple but effective DNAzyme-mediated signal cycle amplification for precise cathodic PEC bioassays.

Porphyrin-based COF (p-COF) has been found to show efficient photoactive and electroactive properties. Each structural unit in the unique long-range ordered periodic p-COF nanocrystals contains abundant active sites and signal generators. This makes p-COF an ideal candidate for the design of PEC biosensors. Li et al. developed a S-scheme PEC immunoassay platform for cardiac troponinI (cTnI) detection based on p-COF and a p-type silicon nanowire array (p-SiNW)-modified photocathode (Figure 3B) [67]. p-SiNW as the photocathode material enhanced the signal-on response with low background and good anti-interference ability. Meanwhile, p-COF modified on p-SiNW enhanced the stability of the sensing electrode and provided abundant sites for the covalent immobilization of anti-cardiac troponin I (anti-cTnI). The resulting S-scheme heterojunctions efficiently improved the separation and transfer rates of the photogenerated carriers due to the well-matched electronic structures of p-SiNW and p-COF. The PEC method achieved the detection of cTnI in human serums with a range of 5 pg/mL–10 ng/mL and a detection limit of 1.36 pg/mL. In comparison with the commercial ELISA, the relative deviation of this method ranged from 0.06 to 0.18% and the recovery rate ranged from 95.4 to 109.5%.

**Figure 4:**
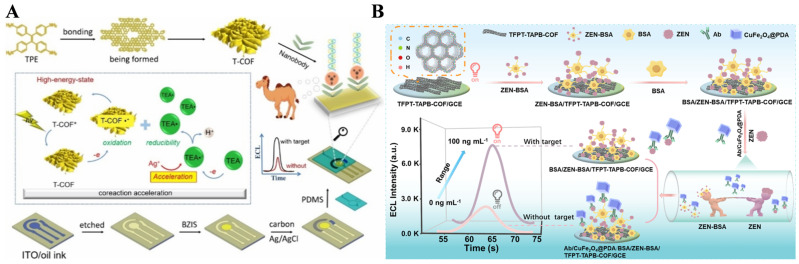
(**A**) Schematic illustration for fabricating TSLP microfluidic immunosensor. Reprinted with permission from ref. [71]. Copyright 2024 American Chemical Society. (**B**) Schematic diagram of the constructed reversal signal “on-off-on” ECL immunosensor based on TFPT-TAPB-COF with the aid of the trimodal strategy for the ZEN detection. Reprinted with permission from ref. [72]. Copyright 2025 Elsevier B.V.

Electrochemiluminescence (ECL) involves the electron transfer reaction of electrically generated species to form excited states of luminescence. It provides temporospatial and spectral resolution as a powerful tool for biosensing by integrating electrochemical and spectroscopic technologies [68]. However, the encapsulation or adsorption of traditional luminophores on the electrode surface may cause unpredictable molecular leakage issues, such as ruthenium complexes, luminol and its derivatives, quantum dots, and carbon nitride nanosheets [69]. In addition, the quenching effect caused by irreversible aggregation of luminophores via π-π stacking interactions may lead to signal attenuation or even disappearance [70]. In contrast, aggregation-induced ECL (AIECL) can enhance the ECL efficiency in the aggregated or solid state but not the dispersed state, which precisely meets the requirement of ECL biosensors. Jia et al. reported a microfluidic ECL immunoassay chip for thymicstromal lymphopoietin (TSLP) detection with tetrakis(4-aminophenyl)ethene (TPE)-derived COF (T-COF) as the ECL emitter (Figure 4A) [71]. A camel-derived nanobody was used as the immune recognition unit. Compared with monoclonal antibodies, the nanobody exhibits higher specificity, residual activity, and epitope recognition ability after solidification. The captured TSLP was recognized by a double-stranded DNA (dsDNA) embedded with Ag^+^ through the Ag–cytosine interaction. In this method, Ag^+^ served as an excellent accelerator to generate free radical species, allowing for the signal-on ECL detection of TSLP in the range of 1 pg/mL to 4.00 ng/mL. The recovery rate was not significantly different from that of ELISA, which is indicative of the high precision and accuracy of this method. The work also suggested that COFs could be utilized to constrain luminescent molecules and trigger the AIECL phenomenon, facilitating the design of novel ECL biosensors. In addition, Li et al. developed a “on-off-on” ECL immunosensor for zearalenone (ZEN) detection using triazine-COF as the luminophore and CuFe_2_O_4_ and polydopamine (CuFe_2_O_4_@PDA) composite as the quencher (Figure 4B) [72]. The triazine-COF was prepared through the reaction between 2,4,6-tris (4-formyl-phenyl)-1,3,5-triazine (TFPT) and 1,3,5-tris(4-aminophenyl)benzene (TAPB). The TFPT-TAPB-COF had the advantages of large specific surface area and pore size, extended π-conjugated skeleton, long-range order structure, donor–acceptor conjugated structure, and high intramolecular charge transfer. These properties can enhance the emission capability of ECL in the presence of K_2_S_2_O_8_. In combination with the trimodal quenching effects of CuFe_2_O_4_@PDA coming from resonance energy transfer, electron transfer, and efficient scavenging ability toward electrogenerated co-reactant radical, the ECL method achieved the competitive immunoassay of ZEN from food products with a detection limit down to 7.9 fg/mL.

**Table 1:** Analytical performances of COF−based electrode materials for immunoassays.

Electrode Material	Analyte	Linear Range	Detection Limit	Ref.
TpBD/Nafion	AFM1	0.5–80 ng/mL	0.15 ng/mL	[45]
Ce-MOF/TPN-COF/CNT	CA125	1 × 10^−4^–100 U/mL	0.088 mU/mL	[47]
COF/MOF-LDH	NfL	5 × 10^−4^–100 ng/mL	17 fg/mL	[48]
MIL156 MOF@COF	CA15-3	30–100 nU/mL	2.6 nU/mL	[49]
m-COF	*E. coli*	10–10^8^ CFU/mL	3 CFU/mL	[50]
COF-LZU1	CA125	0.001–40 U/mL	0.23 mU/mL	[51]
Pt-COFs	CRP	1–400 ng/mL	0.2 ng/mL	[52]
CuS@COFs	AβO	10^−3^–10^3^ nM	0.4 pM	[53]
COFs-AuNPs	KIM-1	0.01–50 pg/mL	2 fg/mL	[54]
Ag_2_O/g-C_3_N_4_-COOH@MA-DBB-COF	AFM1	0.03–1000 fg/mL	0.01 fg/mL	[55]
Au/COF-LZU1	CRP	0.2–20 ng/mL	0.1 ng/mL	[56]
Fe_3_O_4_ NPs@COF/AuNPs	AFP	0.01–1 pg/mL	3.3 fg/mL	[57]
PAF-130	α-Syn	10^−5^–10^3^ ng/mL	3.6 fg/mL	[66]
p-COF@p-SiNW	cTnI	5 × 10^−3^–10 ng/mL	1.36 pg/mL	[67]
T-COF	TSLP	10^−3^–4 ng/mL	2.72 pg/mL	[71]
TFPT-TAPB-COF	ZEN	10^−5^–10^2^ ng/mL	7.9 fg/mL	[72]
Pd/COF-LZU1	CRP	5–180 ng/mL	1.66 ng/mL	[73]
Ru-MCOF	cTnI	10^−6^–10 ng/mL	0.42 fg/mL	[74]
COF-ABEI	cyt c	10^−6^–0.1 ng/mL	0.73 fg/mL	[75]

Abbreviations: TpBD, COF synthesized by triformylphloroglucinol and benzidine. AFM1, alfatoxin M1. Ce-MOF, Cerium-based metal–organic framework. TPN-COF/CNT, terephthalonitrile-based COF and carbon nanotube. CA125, carcinomic antigen 125. COF/MOF-LDH, polyvalent Tb-MOF and CoAl-layered double hydroxide-acceded triazine-COF. NfL, neurofilament light chain. MIL156 MOF@COF, COF-coated MIL-156 MOF. CA15-3, carbohydrate antigen 15-3. m-COF, magnetic COF. *E. coli*, *Escherichia coli O157:H7*. COF-LZU1, COF synthesized by triformylphloroglucinol and 1,4-diaminobenzene. Pt-COFs, platinum nanoparticle modified COF. CRP, C-reactive protein. CuS@COFs, ultrasmall copper sulfide nanoparticle-engineered COF hybrid nanocomposites. AβO, amyloid-β oligomer. AuNPs, gold nanoparticles. KIM-1, kidney injury molecule-1. Ag_2_O/g-C_3_N_4_-COOH@MA-DBB-COF, silver oxide/carboxy-functionalized graphitic carbon nitride@melamine-dibromo butane COF. Au/COF-LZU1, Au nanoparticles-loaded COF-LZU1. AFP, α-fetoprotein. p-COF@p-SiNW, porphyrin-based COF and p-type silicon nanowire arrays. cTnI, cardiac troponin I. PAF-130, a two-dimensional pyrene COF. α-Syn, α synuclein. T-COF, tetrakis(4-aminophenyl)ethene-derived COF. TSLP, thymic stromal lymphopoietin. TFPT-TAPB-COF, COF synthesized by 2,4,6-tris (4-formyl-phenyl)-1,3,5-triazine and 1,3,5-tris(4-aminophenyl)benzene). ZEN, zearalenone. Ru-MCOF, ruthenium-based metal COF. ABEI, N-(4-aminobutyl)-N-ethylisoluminol. cyt c, cytochrome c.

### 2.2. Signal Labels

**Figure 5:**
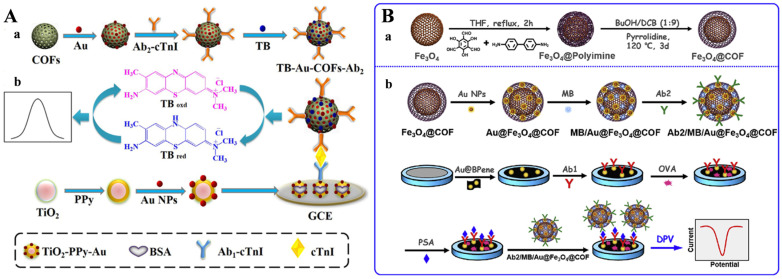
(**A**) Schematic illustration for preparation of TB-Au-COFs-Ab_2_ labels (**a**) sandwich-type electrochemical immunosensor (**b**). Reprinted with permission from ref. [79]. Copyright 2018 Elsevier B.V. (**B**) Schematic illustration for synthesis of Fe_3_O_4_@COF nanohybrid (**a**) and immunoassay of PSA (**b**). Reprinted with permission from ref. [80]. Copyright 2019 Elsevier B.V.

As the signal labels of electrochemical immunoassays, COFs can act as carriers to load redox probes [76,77,78,79,80,81,82], electrocatalysts [83,84,85,86,87,88,89], and enzymes [90,91], or directly serve as redox reporters [92,93,94]. Some organic molecules, such as thionine (Thi), toluidine blue (TB), methylene blue (MB), ferrocene (Fc), and metal complexes, show excellent redox or electrocatalytic properties, which could be embedded in nanocarriers to serve as signal molecules for electrochemical biosensors (Table 2). For example, Zhang et al. reported a sandwich electrochemical immunosensor for cTnI detection using AuNPs-doped TB-loaded COFs (TB-Au-COFs) as signal labels (Figure 5A) [79]. The signal was recorded based on the anodic peak of TB. AuNPs-decorated polypyrrole-modified titanium dioxide nanoparticles (TiO_2_-PPy) were used to immobilize capture antibodies and improve electrode surface area and electronic conductivity. As a result, the target cTnI was determined with a detection limit of 0.17 pg/mL. In addition, Li’s group reported the electrochemical immunoassay of prostate-specific antigen (PSA) using AuNPs-loaded magnetic COFs to load detection antibody and signal molecule MB (Figure 5B) [80]. The AuNPs/black phosphorene (Au@BPene) nanocomposites were modified on the electrode surface to immobilize capture antibodies and improve the electron transfer. The method is very sensitive due to the good electron transfer of Au@BPene, excellent enrichment capacity of MB in COFs, and efficient catalytic activity of Fe_3_O_4_.

**Figure 6:**
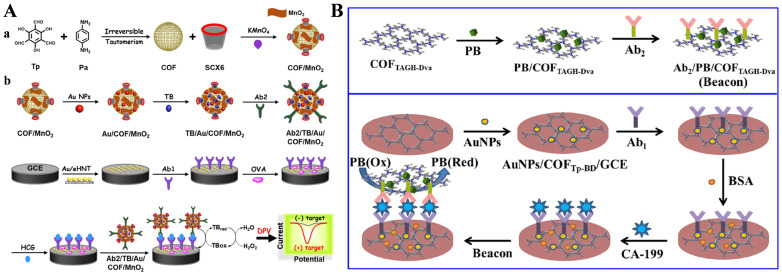
(**A**) Schematic illustration for synthesis of COF/MnO_2_ (**a**) and construction of electrochemical immunoassay (**b**). Reprinted with permission from ref. [83]. Copyright 2021 Elsevier B.V. (**B**) Schematic illustration for electrochemical immunoassay of CA-199. Reprinted with permission from ref. [84]. Copyright 2024 Elsevier B.V.

**Figure 7:**
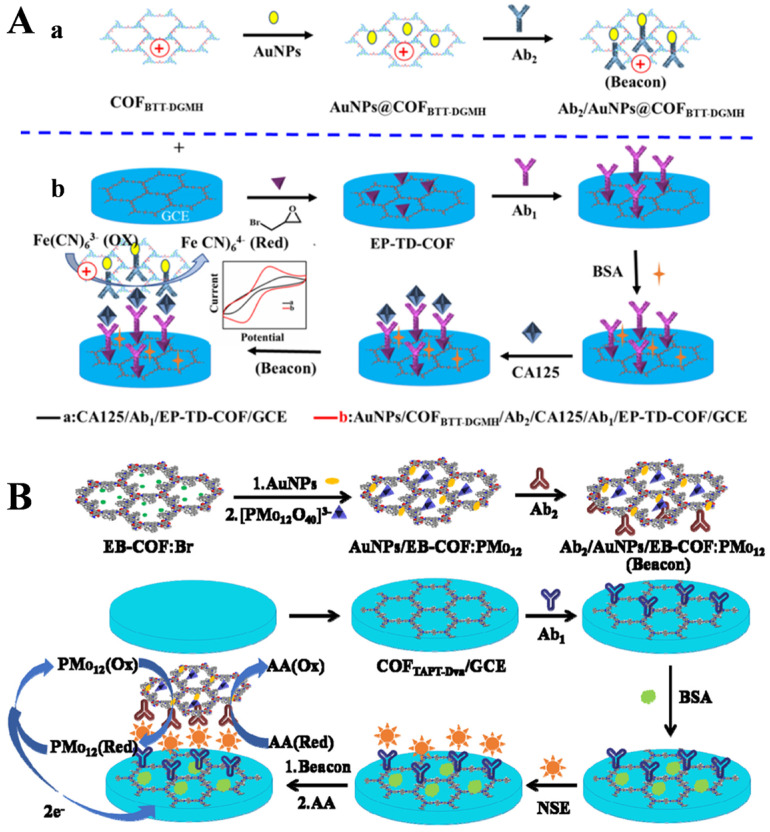
(**A**) Schematic diagram for the construction and application of immunosensor. Reprinted with permission from ref. [86]. Copyright 2025 Elsevier B.V. (**B**) Preparation procedures of the sandwich voltammetric immunosensor. Reprinted with permission from ref. [88]. Copyright 2023 Elsevier B.V.

In addition to being used as carriers to load signal molecules, COFs can also serve as supporters to load electroactive nanoparticles, electrocatalysts, and enzymes for signal amplification. For example, Liang et al. developed a sandwich electrochemical immunosensor for human chorionic gonadotropin (HCG) detection using Au/COF/MnO_2_ composites to immobilize detection antibody Ab_2_ and load signal molecule TB (Figure 6B) [83]. The COF/MnO_2_ was prepared by mixing KMnO_4_, calix[6]arene (SCX6) and COF at room temperature. TB molecules were incorporated into the cavities of SCX6 and the pores of COF via noncovalent interactions. MnO_2_ could catalyze the electrochemical reduction of TB, thus enhancing the signal of the immunoassay. AuNPs were modified on the COF/MnO_2_ to anchor Ab_2_ and further catalyze the reduction of TB. In addition, Wang’s group reported the electrochemical immunoassay of carbohydrate antigen 19–9 (CA 19–9) with COFs to load Prussian blue (PB) (Figure 6A) [84]. The redox signal of Fe^2+^/Fe^3+^ derived from PB in the PB/COF composites increased with the increase in CA 19–9 concentration in the range of 0.01 to 150 U/mL. Jin et al. developed an electrochemical immunosensor for CA125 detection based on the electrostatic interaction between positively charged AuNPs@2DCOFBTT-DGMH and negatively charged [Fe(CN)_6_]^3−/4−^ (Figure 7A) [86]. The epoxy-functionalized EP-TD-COF with large specific surface and full exposure of active sites was modified on the electrode interface to immobilize capture antibody Ab_1_. The electropositive AuNPs@COFBTT-DGMH provided abundant binding sites for the attachment of Ab_2_. More importantly, the hybrids can accelerate the electron transport rate and mass transfer process of [Fe(CN)_6_]^3−/4−^, thereby efficiently amplifying the electrochemical signals. Polyoxometalate consisting of transition metal ions and oxygen atoms have the properties of multi-charges, good stability, and high catalytic activity [95]. PMo_12_ is a common polyoxometalate which has been used in electrochemical sensing fields owing to its good electrocatalytic ability to many substances. For this consideration, PMo_12_ was integrated with AuNPs and COFs to improve the sensitivity of sandwich electrochemical immunoassays based on the electrocatalytic oxidation of ascorbic acid (AA) by PMo12 (Figure 7B) [88]. In this method, electroactive PMo12 was uniformly assembled on AuNPs/COFs composites through an ion-exchanging reaction. AuNPs were grown on the surface of COFs for the immobilization of Ab_2_ via the formation of Au-S bonds.

**Figure 8:**
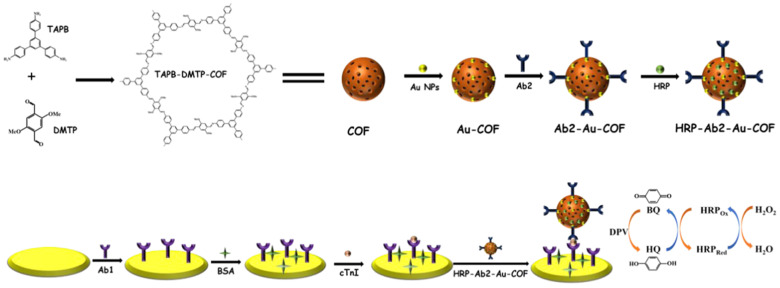
Schematic illustration of the synthesis of HRP-Ab_2_-Au-COF and the constructive procedure of the biosensor for cTnI detection. Reprinted with permission from ref. [90]. Copyright 2021 American Chemical Society.

As natural biocatalysts, enzymes have the properties of high catalytic activity, selectivity, specificity, and biodegradability in various reactions. Thanks to these merits, enzymes are widely used in biosensing fields. However, free enzymes often lack reusability and have many inherent drawbacks, including high cost, low tolerance, and poor stability in harsh environments, hindering their practical applications. Fortunately, numerous studies have shown that immobilization or encapsulation of enzymes by effective strategies is one of the feasible methods to overcome the aforementioned issues [96]. COFs, as the carriers for enzyme encapsulation with high loading capacity, can significantly improve the catalytic performance and reusability of enzymes [97]. For this view, Feng et al. reported an electrochemical immunosensor for cTnI detection using spherical COFs to load the commonly used labeling enzyme horseradish peroxidase (HRP) (Figure 8) [90]. AuNPs were modified on the surface of COFs for the immobilization of detection antibody Ab_2_ and HRP was embedded in the pores of COFs via the host−guest interactions. The signal was produced based on the HPR-catalytic electrochemical oxidation of hydroquinone (HQ) into benzoquinone (BQ) in the presence of H_2_O_2_. The detection limit of this method for cTnI detection was found to be 1.7 pg/mL. This method was successfully put to the real sample assays with good recovery (94.3–104.1%) and reproducibility (relative standard deviation < 4.73%).

**Figure 9:**
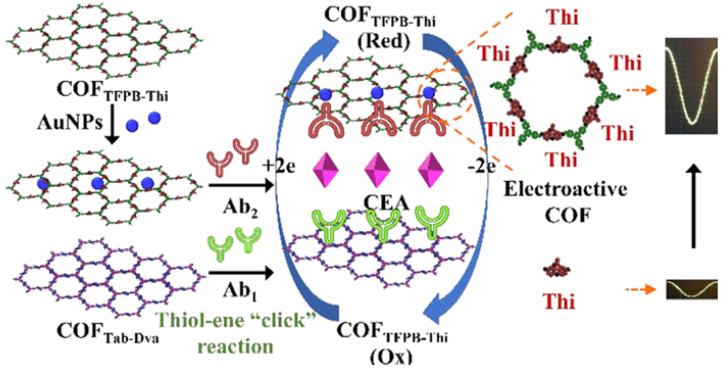
Schematic illustration of the CEA electrochemical immunosensor. Reprinted with permission from ref. [92]. Copyright 2022 American Chemical Society.

**Figure 10:**
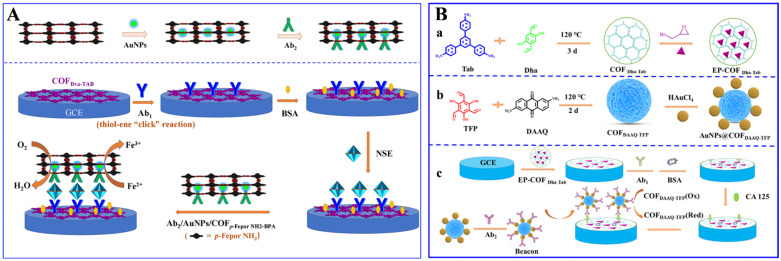
(**A**) Schematic diagram of the NSE immunosensor. Reprinted with permission from ref. [93]. Copyright 2023 Elsevier B.V. (**B**) Schematic illustration of the CA125 electrochemical immunosensor. Reprinted with permission from ref. [94]. Copyright 2025 Elsevier B.V.

The structural diversity of organic molecules can enable the preparation of COFs with specific functional groups according to special requirements. Each structural unit in the long-range ordered periodic nanocrystalline of COFs contains multiple active sites and signal generators. Recently, electroactive COFs have been synthesized and directly exploited as the signal labels of immunoassays by using redox-active organic monomers. For instance, Liang et al. constructed a sandwich electrochemical immunosensing platform using two kinds of two-dimensional COFs as the electrode modifiers and signal labels, respectively. Vinyl-functionalized COF_Tab-Dva_ was modified on the electrode surface to immobilize Ab_1_ by a thiol-ene “click” reaction. Electroactive COF_TFPB-Thi_ was prepared through the acylation reaction between 1,3,5-tris(p-formylphenyl)benzene (TFPB) and thionine, which was then modified with AuNPs to link Ab_2_ for target recognition and signal output (Figure 9) [92]. The method could determine CEA with a detection limit of 0.034 ng/mL. This work presented a novel immunoassay method with electroactive COFs as signal labels. Based on a similar design principle, they also reported sandwich electrochemical immunoassay of neuron-specific enolase (NSE) with iron–porphyrin COFs (COFp-Fepor NH_2_-BPA) as the signal labels (Figure 10A) [93]. The electrochemical signal was monitored by the electrocatalytic reduction of oxygen with COFp-Fepor NH_2_-BPA. In addition, Wu et al. developed a sandwich electrochemical immunosensor with two kinds of COFs as the electrode modifiers and signal labels (Figure 10B) [94]. COF_Dha-Tab_ prepared by 2,5-dihydroxyp-phenyldiformaldehyde (Dha) and 1,3,5-tris(4-aminophenyl)benzene (Tab) was used to absorb capture antibody Ab_1_. Electroactive COF_DAAQ-TFP_ was prepared by the reaction between 2,6-diaminoanthraquinone (DAAQ) and 2,4,6-triformylphloroglucinol (TFP) and then modified with AuNPs for the immobilization of detection antibody Ab_2_. CA125 was specifically captured and detected based on the electrochemical signal of AuNPs@COF_DAAQ-TFP_.

**Figure 11:**
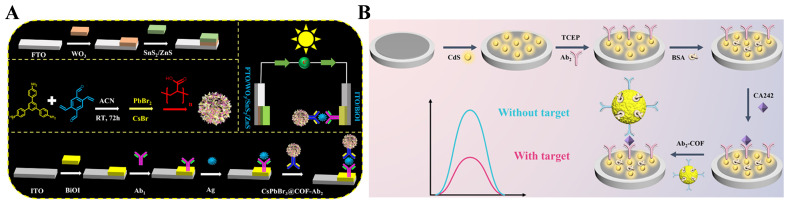
(**A**) Schematic illustration for the fabrication of PEC biosensor. Reprinted with permission from ref. [99]. Copyright 2023 Elsevier B.V. (**B**) Schematic illustration for the fabrication of ECL immunosensor. Reprinted with permission from ref. [100]. Copyright 2024 Royal Society of Chemistry.

Biosensors, by integrating modern optoelectronics and biological systems, have broad prospects. In the immunoassay field, there are few reports on self-powered PEC devices with excellent performances by cathode signal amplification. COFs have excellent biocompatibility and stable storage in solution, providing favorable conditions for their utilization as the carriers of biomolecules [98]. Leng et al. developed a PEC immunoassay device for the detection of heart fatty acid-binding protein (H-FABP) using CsPbBr3@COF-V as the photocathode signal quenching source (Figure 11A) [99]. The self-powered PEC immunosensor was designed with a BiOI photocathode and WO_3_/SnS_2_/ZnS photoanode. The high efficiency and stable self-powered biosensor formed by a self-assembly photoanode heterojunction structure and photocathode on the electrode surface not only provided continuous and powerful photocurrent response for bioanalysis through reasonable stepped band structure, but also effectively eliminated the interference of reducing substances. The quenching source CsPbBr3@COF-V greatly affected the photocurrent response due to steric hindrance, weak conductivity, and competition with the substrate for dissolved oxygen as well as excitation light source. Intervention of these key factors achieved multiple signal amplification effects and opened up an innovative vision for a self-powered PEC immunosensor. H-FABP in the linear range of 0.0005–150 ng/mL was determined with a detection limit of 0.19 pg/mL. In addition, there are few reports on ECL immunoassays with COFs as the signal labels, although COFs show excellent light absorption and have been used for photocatalysis to promote the resonance energy transfer process in ECL. Recently, Han et al. reported a quenching-type ECL immunoassay method for carbohydrate antigen 24-2 (CA242) detection using COFs as the energy acceptors (Figure 11B) [100]. In this work, semiconductor CdS nanocrystals (NCs) with good ECL performance were modified with capture antibody Ab_1_ and used as the energy donors. The ECL signal of CdS NCs was quenched by the detection antibody Ab_2_-modified COFs by resonance energy transfer strategy. This immunosensor can determine CA242 with a detection limit of 0.183 mU/mL.

**Table 2:** Analytical performances of COFs-based signal labels for electrochemical immunoassays.

Signal Label	Analyte	Linear Range	Detection Limit	Ref.
MB@aCOFs-ssDNA	AβO	0.01–1000 nM	5.1 pM	[76]
Thi-Au-COFs	Latexin	0.01–100 ng/mL	50 pg/mL	[77]
Thi/Au/COF	CD44	1–106 pg/mL	0.71 pg/mL	[78]
TB-Au-COFs	cTnI	5 × 10^−4^–10 ng/mL	0.17 pg/mL	[79]
MB/Au@Fe_3_O_4_@COF	PSA	10^−4^–10 ng/mL	30 fg/mL	[80]
TB-Au-COF	CYFRA21-1	0.5–10^4^ pg/mL	0.1 pg/mL	[81]
TB/AuNPs/COF	Apo-A4	0.01–300 ng/mL	2.16 pg/mL	[82]
Tb/Au/COF/MnO_2_	HCG	5 × 10^−4^–10^2^ mIU/mL	1.67 × 10^−4^ mIU/mL	[83]
PB/COF_TAGH-Dva_	CA 19–9	0.01–150 U/mL	0.003 U/mL	[84]
MB/AuPt@MnO_2_@COF	PSA	5 × 10^−5^–10 ng/mL	16.7 fg/mL	[85]
AuNPs@2DCOFBTT-DGMH	CA125	0. 27–10^5^ mU/mL	0.089 mU/mL	[86]
Ti-MOF@COF	GL-3	0.0001–20 ng/mL	0.025 pg/mL	[87]
AuNPs/EB-COF:PMo12	NSE	5 × 10^−5^–10^2^ ng/mL	166 fg/mL	[88]
COF@AuNP	*Salmonella*	2 × 10^2^–2 × 10^5^ CFU mL	60 CFU/mL	[89]
HRP-Au-COF	cTnI	0.005–10 ng/mL	1.7 pg/mL	[90]
COFs-AuNPs-HRP	sPD-L1	0.001–100 ng/mL	0.143 pg/mL	[91]
AuNPs/COFTFPB-Thi	CEA	0.11–80 ng/mL	0.034 ng/mL	[92]
COFp-Fepor NH_2_-BPA/AuNPs	NSE	5 × 10^−4^–10^2^ ng/mL	166.7 fg/mL	[93]
AuNPs/COF_DAAQ-TFP_	CA125	0.01–100 U/mL	6.7 mU/mL	[94]
CsPbBr_3_@COF–V	H-FABP	0.0005–150 ng/mL	0.19 pg/mL	[99]
COF_TAPB_-_DMTP_	CA242	0.001–1000 U/mL	0.183 mU/mL	[100]

Abbreviation: MB, methylene blue. aCOFs, alkynyl COFs. AβO, amyloid-β oligomer. Thi, thionine. CD44, a potential biomarker for breast cancer. TB, toluidine blue. cTnI, cardiac troponin I. PSA, prostate-specific antigen. CYFRA21-1, cytokeratin fragment antigen 21-1. AuNPs, gold nanoparticles. Apo-A4, apolipoprotein A4. HCG, human chorionic gonadotropin. PB, Prussian blue. CA 19–9, carbohydrate antigen 19–9. MnO_2_, manganese dioxide. 2DCOFBTT-DGMH, 2D COF prepared by benzotrithiophene tricarbaldehyde and 1,3-diaminoguanidine monohydrochloride. CA125, cancer antigen 125. MOF, metal–organic framework. GL-3, galectin-3. PMo12, phosphomolybdic acid H3[PMo12O40]. NSE, neuron-specific enolase. HRP, horseradish peroxidase. sPD-L1, soluble programmed death-ligand 1. TFPB, 1,3,5-tris(p-formylphenyl)benzene. CEA, carcinoembryonic antigen. COF_DAAQ-TFP_, COF synthesized by 2,6-diaminoanthraquinone and 2,4,6-triformylphloroglucinol. COFp-Fepor NH_2_-BPA, iron–porphyrin COF. H-FABP, heart fatty acid-binding protein. COF_TAGH-Dva_, vinyl-rich COF prepared by the reaction between triaminoguanidine hydrochloride and 1,4-benzenedicarboxaldehyd. COF_TAPB_-_DMTP_, COFs prepared from TAPB and DMTP. CA242, carbohydrate antigen 24-2.

## 3. COFs-Based Optical Immunoassays

**Figure 12:**
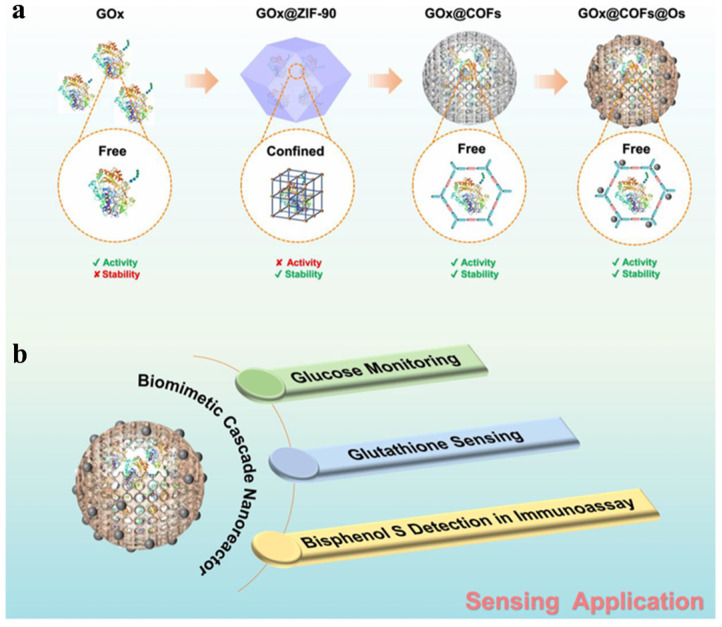
Synthetic illustration of biomimetic cascade nanoreactor (GOx@COFs@Os) capsule (**a**) and biosensing applications (**b**). Reprinted with permission from ref. [104]. Copyright 2023 American Chemical Society.

**Figure 13:**
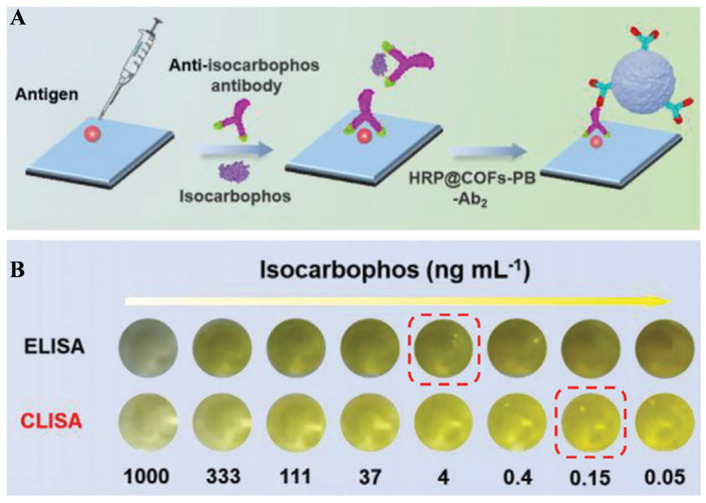
(**A**) Schematic illustration of HRP@COFs-PB-based CLISA for imidacloprid detection. (**B**) Color change of HRP-COFs composite-based CLISA with the increase of isocarbophos concentration. Reprinted with permission from ref. [105]. Copyright 2025 Wiley-VCH.

Optical immunoassays are a category of biosensors that utilize optical signals for the detection and quantification of antigens or antibodies. Typically, optical immunoassays employ signal labels, such as colorimetric, fluorescent, chemiluminescent, and SERS molecules or materials (Table 3), to respond to immunoreactions [101,102]. Among them, colorimetric immunoassays have attracted considerable attention owing to their high simplicity and efficiency [103]. Traditional colorimetric immunological analysis methods mainly include ELISA and lateral flow immunoassay (LFIA). In the former, natural enzymes and nanozymes are the main reporters for direct or indirect signal amplification. However, natural enzymes face significant challenges as signal amplifiers due to their low stability and poor catalytic activity under harsh conditions. Coupling enzymes with scaffolds instead of directly labeling enzyme molecules can maintain the integrity of enzyme structures and improve the analysis stability of enzymatic signal amplification methods. COFs are believed to be ideal materials for enzyme immobilization owing to their features such as well-defined channels, tunable pore environment, robust reticular architecture, and tailored functionality. Wei et al. suggested that a natural enzyme (glucose oxidase, GOx) and synthetic catalyst (Os nanozyme) can be encapsulated and immobilized on COFs to trigger a catalytic cascade reaction for bioassays (Figure 12) [104]. ZIF-90 MOF was employed as the template for the formation of the COF capsule and the encapsulation of GOx. Os nanozyme was formed on the COF capsule to improve the affinity and diffusion efficiency of the substrate due to the good pore structure. The resulting GOx@COFs@Os capsule promoted the tolerance of GOx to harsh environments and improved the conformational freedom of GOx for maintaining its activity. The catalytic efficiency was improved by 2.19-fold compared to the free cascade system. The boosted biocatalytic performance facilitated the detection of glucose and glutathione. The GOx@COFs@Os probe has also been used for sandwich immunoassay of bisphenol S (BPS) on a microplate with a detection limit of 0.038 ng/mL that is 5.66 times higher than that of the traditional ELISA. In addition, Yan et al. suggested that enzymes can be assembled in situ into the framework structure during the crystal formation process of COFs (Figure 13) [105]. The enzyme–COFs–PB composites were formed by the condensation between p-phenylenediamine and benzene-1,3,5-tricarboxaldehyde in the presence of HRP. In the process, the structure and activity of HRP were well maintained after encapsulation in COFs-PB, and the HRP@COFs-PB composites showed outstanding stability in a strongly acidic environment. The immunosensor named CLISA was used for the determination of isocarbophos with a 56-fold enhancement in sensitivity compared with that of the standard ELISA method.

**Figure 14:**
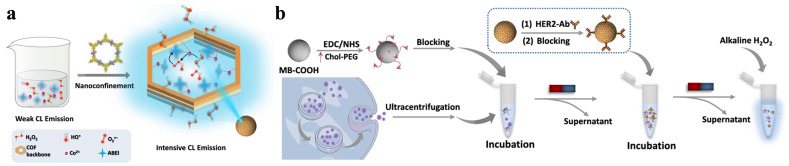
Schematic illustration of the confinement-enhanced CL mechanism (**a**) and the lipid–protein dual-recognition sandwich strategy for EVs based on CAACo (**b**). Reprinted with permission from ref. [106]. Copyright 2023 American Chemical Society.

Chemiluminescence (CL) is an emission phenomenon induced by chemical reaction. Lyu et al. reported a CL immunoassay method by the co-embedding of N-(aminobutyl)-N-(ethyl isoluminol) (ABEI) and Co^2+^ into AuNP-modified COFs (named CAACo) (Figure 14) [106]. A COF synthesized by the condensation between 2,5-divinyltereph thalaldehyde and 1,3,5-tris(4-aminophenyl)benzene provided abundant aromatic groups and imine groups as binding sites for ABEI and Co^2+^ embedment through π-π stacking and coordination interactions. The confinement of ABEI and Co^2+^ into Au-COF achieved at least a 20-fold enhancement of CL intensity compared to adding them to a liquid phase. The CAACo nanomaterial was used as the signal tag for a confinement-enhanced CL immunoassay of breast cancer cell line-derived extracellular vesicles (EVs) with a detection limit of 2.6 × 10^5^ particles/mL. In contrast to other CL-functionalized materials, the superiority of CAACo was not only reflected in the intensive emission but also in its convenient conjugation with antibodies, providing a new perspective for the development of CL biosensors.

**Figure 15:**
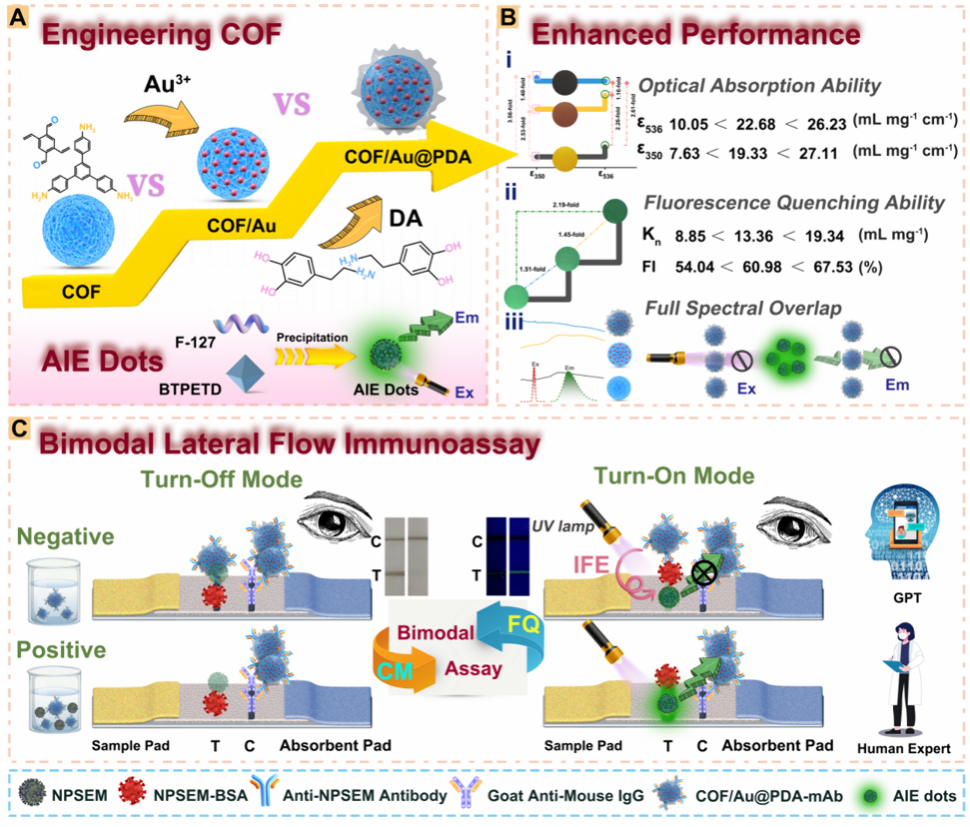
(**A**) Schematic illustration for engineering COF and AIE dots. (**B**) Enhanced performances for COF/Au@PDA. (**C**) Bimodal assay of COF/Au@PDA-LFIA. Reprinted with permission from ref. [108]. Copyright 2024 American Chemical Society.

LFIA is highly valued in the field of point-of-care testing due to its advantages of fast detection, low cost, and convenient operation. In the LFIA method, a signal is usually generated through immunoreactions between probe, analyte, and antibody. However, the traditional LFIA method with colloidal gold as the color indicator suffers from low sensitivity, limiting its application in the early diagnosis of diseases [107]. Therefore, novel signal labels and detection techniques have been developed to amplify the colorimetric, fluorescence, SERS, or electrochemical signals in LFIA methods. For example, Cheng et al. developed a colorimetric–fluorescence bimodal LFIA method using AuNPs and a polydopamine (PDA)-engineered COF named COF/Au@PDA as the fluorescence quencher and colorimetric indicator (Figure 15) [108]. In this method, COF endowed the COF/Au@PDA label with excellent optical properties, outstanding fluorescence quenching ability, and good water dispersibility. Owing to the excellent visible light absorption of a COF with a donor−acceptor structure, localized surface plasmon resonance (LSPR) ability of AuNPs, and broad light absorption of the PDA layer, the COF/Au@PDA exhibited strong absorption and full spectrum overlap towards AIE dots. The detection limit of fluorescence-quenching LFIA (named FQ-LFIA) is six-fold lower than that of colorimetric LFIA (named CM-LFIA). In addition, carbon dots (CDs) as fluorescence donors and COFs as fluorescence acceptors have been used for the immunoassay of *Escherichia coli O157: H7* (*E. coli O157:H7*) through fluorescence resonance energy transfer (FRET) [109]. The fluorescence biosensor had a linear detection range of 0−10^6^ CFU/mL and a detection limit of 7 CFU/mL.

**Figure 16:**
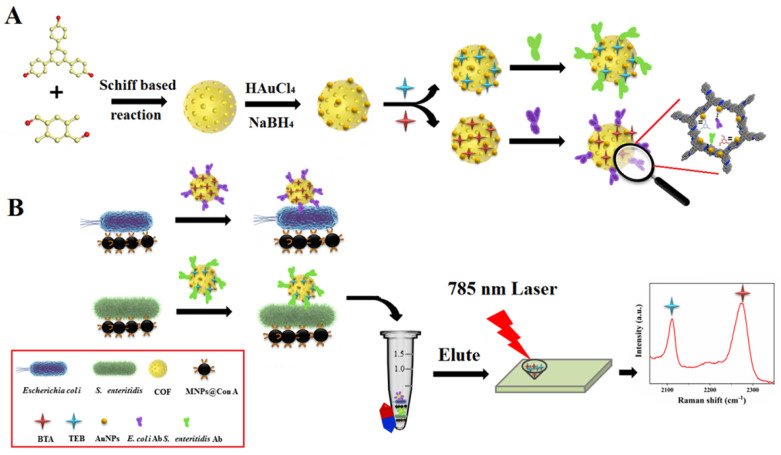
(**A**) Schematic illustration of the preparation procedure of novel COFs-based Raman tags and the interaction between TBDP and Raman reporters/antibody. (**B**) Schematic illustration of simultaneous immuno-SERS detection of *E. coli* and *S. enteritidis*. Reprinted with permission from ref. [111]. Copyright 2022 Elsevier B.V.

SERS is a highly sensitive technique that can enhance the Raman scattering of molecules supported by certain nanostructured materials. It can allow for structural fingerprinting of low concentration analytes through plasma-mediated amplification of electric fields or chemical enhancement. Due to its high sensitivity and selectivity, SERS has a wide range of applications in surface and interface chemistry, catalysis, nanotechnology, biology, biomedicine, food science, environmental analysis, and other fields [110]. Recently, there have been limited reports on the application of COFs for SERS biosensors. For example, a SERS immunosensor has been developed and used for multiple detection of foodborne pathogens with multifunctional COFs as the Raman tags (Figure 16) [111]. In this method, magnetic nanoparticles (MNPs) modified with concanvilin A (Con A) were employed to capture foodborne pathogens. TBDP COFs prepared from 1,3,5-tris(4-aminophenyl)benzene and 2,5-dimethoxyterephaldehyde were coated with AuNPs for loading Raman reporters and antibodies. After sandwich immunoreaction and magnetic separation, Raman reporters were released from the immunocomplexes by the eluent of N,N-Dimethylformamide (DMF) and then determined under 785 nm laser beams. Two different foodborne pathogenic strains have been simultaneously determined with an equal detection limit of 10 CFU/mL according to the characteristic Raman signals of the reporters at 2271 and 2113 cm^−1^. The strategy, providing new insight into the application of SERS, can be used for the design of different multiplex SERS immunosensing platforms by changing the Raman tags.

**Figure 17:**
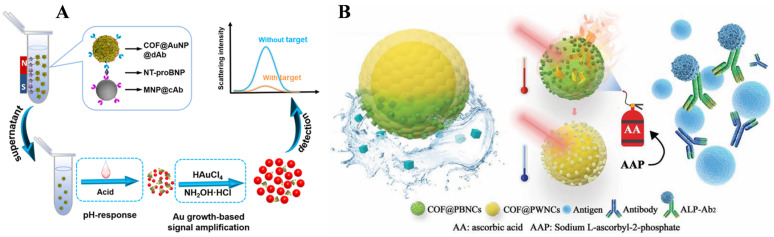
(**A**) Schematic illustration of the preparation procedure of novel COFs-based Raman tags and the interaction between TBDP and Raman reporters/antibody. Reprinted with permission from ref. [113]. Copyright 2022 Elsevier B.V. (**B**) Schematic illustration of simultaneous immuno-SERS detection of *E. coli* and *S. enteritidis*. Reprinted with permission from ref. [114]. Copyright 2023 Wiley-VCH.

Dynamic light scattering (DLS) is a technique that can measure the size distribution of particles in solution. It is considered an attractive alternative to improve the sensitivity of immunoassays due to its advantages of high sensitivity, simple operation, and fast data collection [112]. However, the sensitivity of traditional DLS immunosensors is far from satisfactory to measure the low concentration of targets based on limited size change. To improve the sensitivity, Guo et al. reported a DLS immunoassay method by combining COF@AuNP heterostructures with a gold growth technique (Figure 17A) [113]. COF@AuNP captured by the sandwich immunoreactions could release a large number of AuNPs under an acidic condition. Then, the released AuNPs were enlarged by gold growth to produce an amplified DLS signal, thereby remarkably improving the sensitivity of immunoassays.

Photothermal materials are widely popular in the field of point-of-care testing. COFs have been used as substrates to load photothermal nanomaterials for bioassays. For example, COF nanoparticles were prepared by the reaction between 1,3,5-tris(4-aminophenyl)benzene (TPB) and 2,5-divinylterephthalaldehyde (DVA) and used for the in situ growth of photothermal materials, Prussian blue nanocubes (PBNCs) (Figure 17B) [114]. The COF@PBNCs were used as the probes for the immunoassay of furosemide in a competitive format. In this work, ascorbic acid generated by the alkaline phosphatase-catalyzed hydrolysis of AAP (sodium L-ascorbyl-2-phosphate) reduces PBNCs into PWNCs (Prussian white nanocubes). This made COF@PBNCs lose photothermal activity, providing a new insight into the development of photothermal point-of-care testing.

**Table 3:** Analytical performances of COFs-based optical immunoassays.

Method	Material	Analyte	Linear Range	Detection Limit	Ref.
Color	GOx@COFs@Os	BPS	0−1.6 ng/mL	0.038 ng/mL	[104]
Color	HRP@COFs-PB	Isocarbophos	0.05–1000 ng/mL	0.03 ng/mL	[105]
CL	CAACo	EVs	3.3 × 10^5^–3.3 × 10^8^ particles/mL	2.6 × 10^5^ particles/mL	[106]
CM-FQ	COF/Au@PDA	NPSEM	0.1–1.5 ng/mL, 0.1–1.2 ng mL	0.1 ng/mL, 0.6 ng/mL	[108]
FRET	CD@COF	*E. coli O157:H7*	0–10^6^ CFU/mL	7 CFU/mL	[109]
SERS	TBDP@Au	*E. coli*, *S. enteritidis*	10^2^–10^4^ CFU/mL	10 CFU/mL	[111]
DLS	COF@AuNP	NT-proBNP	0.32–1000 pg/mL	14 fg/mL	[113]
Photothermal	COF@PBNCs	Furosemide	0.05–100 ng/mL	10.6 pg/mL	[114]

Abbreviation: GOx, glucose oxidase. Os, osmium nanozyme. BPS, bisphenol S. COFs-PB, covalent organic frameworks synthesized by the condensation between p-phenylenediamine and benzene-1,3,5-tricarboxaldehyde. CL, Chemiluminescence. CAACo, N-(aminobutyl)-N-(ethyl isoluminol)/Co^2+^-embedden gold nanoparticle-modified COFs. EVs, extracellular vesicles. COF/Au@PDA, gold nanoparticle/polydopamine-coengineered COFs. CM-FQ, colorimetric and fluorescence-quenching lateral flow immunoassays. NPSEM, a furacillin metabolite. FRET, fluorescence resonance energy transfer. SERS, Surface Enhanced Raman Scattering. TBDP, COFs prepared by 1,3,5-tris(4-aminophenyl)benzene and 2,5-dimethoxyterephaldehyde. *E. coli O157:H7*, *Escherichia coli O157: H7*. *E. coli*, *Escherichia coli* ATCC25922. *S. enteritidis*, *Salmonella enteritidis*. DLS, dynamic light scattering. NT-proBNP, N-terminal brain-type natriuretic peptide. PBNCs, Prussian blue nanocubes.

## 4. Conclusions

The superior advantages of COFs, such as large surface area, high thermal and chemical stability, structural flexibility, and abundant functional groups, endow them with great potential in the development of sensing devices. COFs with specific functions can be precisely synthesized by selecting functional organic units (e.g., optically active fragments, redox-active centers, or catalytic units). In this review, the advance of electrochemical and optical immunoassays based on COFs and their hybrids has been comprehensively summarized. In order to further clarify the detection mechanism, some typical and important works are highlighted and discussed in detail. The detection performances of various COFs-based immunoassays are shown in Table 1, Table 2 and Table 3, and the synthesis conditions for the used COFs are shown in Table 4.

Although a series of highly sensitive and selective COFs-based immunoassays have been successfully developed, there are still several challenges to be addressed. First, most COFs are synthesized through solvothermal methods, which have the limitations of high reaction temperature, long reaction time, toxic organic solvent, and complex reaction processes. Therefore, it is urgent to explore simpler and greener synthesis methods for the large-scale production of COFs, such as room temperature vapor-assisted conversion, self-exfoliation, and solvent-assisted exfoliation for two-dimensional COFs, as well as solvothermal synthesis, mechanochemical synthesis, and room-temperature synthesis for three-dimensional COFs. Second, the relationship between the structural characteristics of COFs and their sensing performances should be systematically studied to guide the successful preparation of COFs, such as building blocks, linkage bonds, topological structure, size, porosity, and conductivity. Most COFs exhibit intrinsic shortcomings, such as poor conductivity and relatively low fluorescence quantum yields, which limit their application in electrochemical and optical immunoassays. Screening more novel building blocks with luminescence or completely *π*-conjugated planar structures may be an alternative approach to prepare COFs with excellent optical or electric properties. In addition, COFs with large surface areas and pores can serve as carriers to load other functional materials or molecules. The rational combination of them can overcome individual defects, generate new properties, and improve detection performance via synergetic effects. For instance, the introduction of conductive materials can greatly enhance the conductivity of pure COFs. Third, the efficient and controllable immobilization of antibodies on COFs and their hybrids play an important role in the reproducible and accurate preparation of immunosensors. More innovative strategies for oriented immobilization should be designed by considering the physicochemical and chemical properties of COFs and biomolecules. Undoubtedly, immunoassays based on COFs and their hybrid materials have great research potential and application prospects. Significant achievements can be made through the joint efforts of multiple research fields, including materials science, nanotechnology, analytical chemistry, and biological science. In addition, the screening of experimental conditions and structure–property relationships can be explored through computational calculations and automated machine learning which are becoming powerful tools for obtaining tailored materials.

## Figures and Tables

**Table 4:** Reagents and reaction conditions for the synthesis of COFs used in immunoassays.

COF Name	Reagent	Reaction Condition	Ref.
COF_Tp-BD_	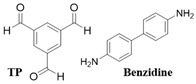	120 °C for 3 days in the mixed solvent of mesitylene and dioxane with acetic acid as catalyst [115]	[45,49,80,111,116]
COF-LZU1	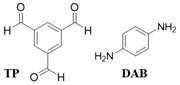	120 °C for 3 days in 1,4-dioxane with acetic acid as catalyst [117]	[46,51,52,56,75,79,89,105,113]
triazine-COF	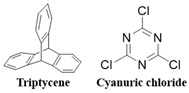	40 °C for 16 h in dichloromethane with AlCl_3_ as catalyst [118]	[48]
TPN-COF	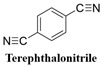	400 °C for 5 h with ZnCl_2_ as catalyst [119]	[47]
COF_TAPB_-_DMTP_	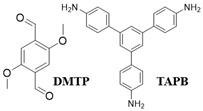	70 °C for 24 h in the mixed solvent of *n*- butanol and 1,4 ethylene oxide with acetic acid as catalyst	[53,57,58,77,90,100,120]
COF_Dha-Tab_	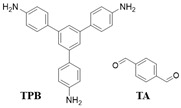	25 °C for 45 min in DMSO with acetic acid as catalyst	[54,91]
MA-DBB	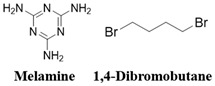	150 °C for 2 h in DMSO	[55]
p-COF	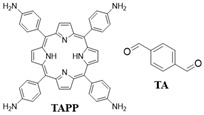	90 °C for 14 h in the mixed solvent of o-dichlorobenzene and 1-butanol with acetic acid as catalyst	[67]
PAF-130	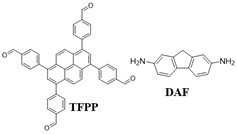	120 °C for 3 days in the mixed solvent of o-dichlorobenzene and n-butanol with acetic acid as catalyst	[66]
T-COF	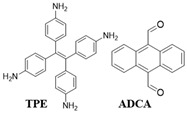	120 °C for 96 h in the mixed solvent of mesitylene and dioxane with acetic acid as catalyst	[71]
TFPT-TAPB-COF	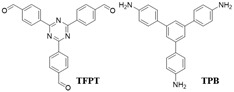	120 °C for 3 days in the mixed solvent of 1,4-dioxane and mesitylene with acetic acid as catalyst [121]	[72]
Ru-MCOF	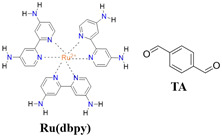	120 °C for 3 days in the mixed solvent of o-dichlorobenzene and ethanol with acetic acid as catalyst	[74]
m-COF	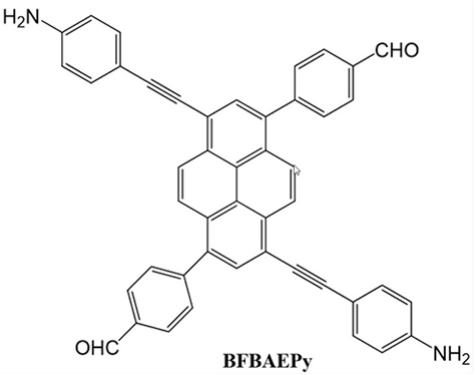	120 °C for 3 days in THF with acetic acid as catalyst	[50]
COF–V	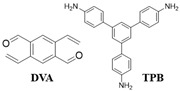	25 °C for 72 h in acetonitrile with acetic acid as catalyst [122]	[99,106,108]
COF	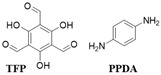	120 °C for 3 days in the mixed solvent of mesitylene and dioxane with acetic acid as catalyst [123]	[83]
pCOF	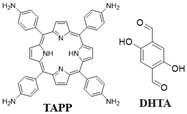	120 °C for 3 days in the mixed solvent of o-dichlorobenzene and 1-butanol with acetic acid as catalyst	[76]
COF_TAGH-Dva_	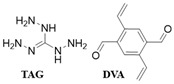	120 °C for 3 days in the mixed solvent of mesitylene and dioxane with acetic acid as catalyst	[84,88]
TFPB-COF	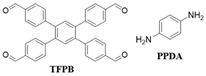	120 °C for 3 days in the mixed solvent of mesitylene and dioxane with acetic acid as catalyst [124]	[85,92]
COFBTT-DGMH	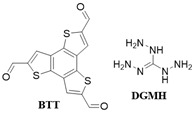	120 °C for 3 days in the mixed solvent of acetone and of 1,4-dioxane with acetic acid as catalyst	[86]
COF	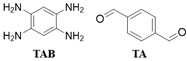	25 °C for 30 min in the mixed solvent of 1,3,5-trimethylbenzene and dioxane with Scandium (III) triflate as catalyst	[78]
EB-COF:Br	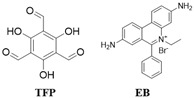	35 °C for 10 days in the mixed solvent of dichloromethane and water with amine-p-toluene sulfonic acid as catalyst [125]	[88]
bipyridine-COF	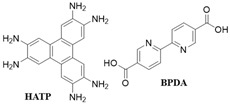	150 °C for 24 h and then 180 °C for 24 h with polyphosphoric acid as solvent and catalyst	[82]
COF_DAAQ-TFP_	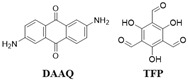	120 °C for 3 days in 1,4-dioxane with acetic acid as catalyst [126]	[94]
COF_p-Fepor_ NH2-BPA	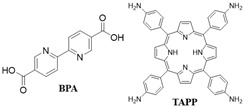	120 °C for 3 days in the mixed solvent of mesitylene and dioxane with acetic acid as catalyst	[93]
COF_Dva-TAB_	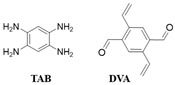	120 °C for 3 days in the mixed solvent of mesitylene and dioxane with acetic acid as catalyst	[93,114]

Abbreviation: TP, triformylphloroglucinol. DAB, 1,4-diaminobenzene. DMTP, 2,5-dimethoxyterephthalaldehyde. TPB, 1,3,5-tris(4-aminophenyl)benzene. TFPP, 1,3,6,8-Tetra(4-formylphenyl)pyrene. DAF, 2,7-diaminofluorene. TPB, 1,3,5-tris(4-aminophenyl)benzene. Ru(dbpy), tris(4,4′-diamino-2,2′-bipyridine) ruthenium (II). BFBAEPy, 1,6-bis(4-formylphenyl)-3,8-bis((4-aminophenyl) ethynyl)) pyrene. TFP, (2,4,6-trihydroxybenzene-1,3,5-tricarbaldehyde). PPDA,1,4-diaminobenzene. TAPP, 5,10,15,20-tetrakis(4-aminophenyl) porphyrin. TA, terephthalaldehyde. TPE, Tetrakis(4-aminophenyl)ethene. ADCA, 9,10-anthracenedicarboxaldehyde. TFPT, 2,4,6-tris (4-formyl-phenyl)-1,3,5-triazine). TAG, triaminoguanidine. DVA, 2,5-divinylterephthalaldehyde. TFPB, 1,2, 4,5-tetrakis-(4-formylphenyl)benzene. DHTA, 2,5-dihydroxyterephthaldeyde. BTT, benzotrithiophene tricar-baldehyde, DGMH, 1,3-diaminoguanidine monohydrochloride. TAB, 3,3′-diaminobenzidine. EB, ethidium bromide. HATP, 2,3,6,7,10,11-hexaaminotriphenyl hexahydrochloride. BPDA, 2,2′-bipyridine-5,5′-dicarboxylic acid. DAAQ, 2,6-diaminoanthraquinone. BPA, 2,2′-bipyridine-5,5′-dicarboxylic acid.

## Data Availability

Not applicable.
